# Exposure to intimate partner violence and subsequent substance use among a nationwide sample of LGBTQIA+ people: results of The PRIDE Study

**DOI:** 10.1093/abm/kaaf091

**Published:** 2025-01-04

**Authors:** Nicholas Metheny, Nguyen Khai Tran, Gabriel John Dusing, Dalton Scott, Micah E. Lubensky, Mitchell R. Lunn, Juno Obedin-Maliver, Annesa Flentje

**Affiliations:** 1 Nell Hodgson Woodruff School of Nursing, Emory University, Atlanta, GA 30322, United States; 2 MAP Center for Urban Health Solutions, Li Ka Shing Knowledge Institute, St Michael’s Hospital, Toronto, ON M5B 1W8, Canada; 3 The PRIDE Study/PRIDEnet, Stanford University School of Medicine, Palo Alto, CA 94305, United States; 4 Department of Obstetrics and Gynecology, Stanford University School of Medicine, Stanford, CA 94305, United States; 5 University of Miami School of Nursing and Health Studies, Coral Gables, FL 33146, United States; 6 Department of Community Health Systems, School of Nursing, University of California, San Francisco, San Francisco, CA 94158, United States; 7 Stanford Prevention Research Center, Department of Medicine, Stanford University School of Medicine, Palo Alto, CA 94305, United States; 8 Department of Epidemiology and Public Health, School of Medicine, Stanford University, Palo Alto, CA 94305, United States; 9 Alliance Health Project, Department of Psychiatry, School of Medicine, University of California, San Francisco, CA 94143, United States

**Keywords:** intimate partner violence, LGBTQIA+, substance use, minority stress

## Abstract

**Background:**

Individuals who are lesbian, gay, bisexual, transgender, queer or questioning, intersex, aromantic, asexual, or another sexual or gender minority (LGBTQIA+) are at greater risk of both intimate partner violence (IPV) and substance use compared to their cisgender, heterosexual counterparts. However, knowledge regarding the complex relationship between IPV and substance use in LGBTQIA+ communities is limited.

**Methods:**

This study used data from 2 years of The PRIDE (Population Research in Identity and Disparities for Equality) Study, a nationwide, community-based sample of LGBTQIA+ adults in the United States. Past-year IPV in 2021 was measured using the Extended-Hurt, Insulted, Threaten, Scream scale. Substances used in 2022 were measured via the NIDA-Modified Alcohol, Smoking, and Substance Involvement Screening Test (NM ASSIST). Linear and modified Poisson regression models explored temporal relationships between past-year IPV and prospective substance use.

**Results:**

Participants (*n* = 3745) were relatively young (median= 34 years, interquartile range: 27.6–48.5) and represented diverse LGBTQIA+ subcommunities. Approximately one-quarter (23.7%) were cisgender women and 17% were cisgender men. Half (49.2%) were gender minority people. Overall, one-quarter (24.6%) reported exposure to IPV in 2021. In adjusted models, past-year IPV was associated with prospective substance use (risk ratio [RR]: 1.11; 95% CI, 1.03–1.19). Additionally, more frequent IPV was associated with a higher prospective NM ASSIST score for cannabis (*B*: 0.22; 95% CI, 0.08–0.36), stimulants (*B*: 0.20; 95% CI, 0.06–0.35), hallucinogens (*B*: 0.07; 95% CI, 0.02–0.12), and narcotics (*B*: 0.12; 95% CI, 0.03–0.20).

**Conclusions:**

Exposure to IPV in 2021 was linked to greater substance use in 2022. Findings underscore the role of IPV in substance use among LGBTQIA+ people. Furthermore, they point to the need for increased IPV screening and referral among LGBTQIA+ people, as well as the potential efficacy of trauma-informed substance use interventions that address IPV.

## Lay Summary

This study explored how experiences of relationship violence may affect future substance use among LGBTQIA+ (lesbian, gay, bisexual, transgender, queer or questioning, intersex, aromantic, asexual, or other sexual and gender minority) adults in the United States. Using data from over 3700 people who participated in a national health study, we looked at whether those who experienced intimate partner violence (IPV)—including emotional, physical, or sexual harm from a romantic partner—were more likely to use substances the following year. About 1 in 4 participants reported experiencing IPV in the past year. Our results showed that people who experienced IPV were more likely to use substances such as cannabis, stimulants, and opioids in the year that followed. We also found that, the more frequent the IPV, the greater the risk of future substance use. These findings suggest that IPV is not only harmful on its own but may also contribute to increased substance use in LGBTQIA+ communities. The results highlight the need for services that are both trauma-informed and LGBTQIA+-affirming, and that address relationship violence as part of substance use prevention and care. Efforts to support LGBTQIA+ people should include screening for IPV and connecting individuals to safe, affirming resources.

## Introduction

Intimate partner violence (IPV) is a serious public health issue that includes aggressive, abusive, controlling, and/or coercive behaviors perpetrated by a current or former intimate partner against another.^1^ Individuals who are lesbian, gay, bisexual, transgender, queer, intersex, aromatic, asexual, or other sexual and gender minority (LGBTQIA+) in the United States have higher rates of IPV victimization than their cisgender, heterosexual counterparts. This is due, in large part, to sexual and gender minority stress, or the impact of ongoing and historical stigma and discrimination faced by LGBTQIA+ communities.^2–4^ Such structural stressors can influence individual IPV victimization risk by increasing internalized and anticipatory stigma in romantic partners,^5^ which can heighten responses such as rejection sensitivity and increase aggression^6^ and IPV.^7^ Minority stressors can also increase IPV victimization by increasing negative self-schemas and self-devaluation in individuals, who may then feel they “deserve” violent behaviors from romantic partners.^8^ Within the LGBTQIA+ community, gender diverse individuals experience IPV at rates 2 to 3 times higher than cisgender people,^9^ and cisgender, bisexual people (regardless of gender) often report significantly higher rates of IPV victimization than heterosexual individuals.^10–12^ Studies found that cisgender gay men and lesbian women have a significantly higher prevalence of IPV compared to cisgender, heterosexual individual.^13–15^ Notably, while the NISVS queried respondents about transgender identity (but not other gender-expansive identities), the authors did not report IPV prevalence estimates for this group as individuals identifying as transgender constituted 0.2% of the study sample. Indeed, there remains a lack of studies examining the prevalence of IPV among gender-diverse individuals.^9^

One reason for these subgroup differences may lie in the different structural stressors faced by members of LGBTQIA+ subcommunities. Informed by an intersectionality framework,^16^ LGBTQIA+ people at the confluence of axes of marginalization (eg, minoritized gender identity and race) experience unique stressors that compound the risk of IPV^17,18^ through the increased vulnerability to maladaptive dyadic processes,^19^ barriers to healthcare access, and diminished opportunities to seek help or exit an abusive relationship.^20^ Unfortunately, much of the IPV literature within the LGBTQIA+ community either focuses on a single subcommunity (eg, cisgender gay men) or collapses all subcommunities into a single “LGBT+” category.^18^ This obscures important differences within the LGBTQIA+ community as well as the potential for differential drivers and opportunities for intervention among subcommunities. Similarly, little research has examined whether different typologies of IPV victimization may have different effects on health outcomes, including substance use, in LGBTQIA+ populations. One large study of LGBTQIA+ adolescents found teens age 13–17 who were sexually harassed and bullied as well as those who experienced sexual victimization, bullying, and sexual harassment were more likely to report poor mental health and substance use than those without victimization.^13^ However, this is not specific to IPV, or the forms of violence measured in this analysis. Evidence in cisgender, heterosexual women suggests that emotional or psychological violence can be as harmful to mental^14,15^ and physical^21^ health as physical IPV. However, these relationships remain largely untested in adult LGBTQIA+ samples.

Substantial evidence shows a complex relationship between IPV and substance use,^22,23^ which is further complicated in LGBTQIA+ communities given they contend with higher rates of both compared to their cisgender and heterosexual counterparts. A 2015 study based on a nationally representative sample of over 34 000 adults in the United States found that gay and bisexual men who experienced past homophobic or biphobic discrimination were significantly more likely (41.9% vs 15.5%) to report lifetime drug use disorder compared to those without such experiences.^24^ A 2024 Canadian study based on linked longitudinal data found that bisexual women had over 3 times higher risk of hospitalizations and deaths due to substances compared to heterosexual women.^25^ Finally, research consistently demonstrates that gender diverse communities experience higher rates of substance use than their cisgender peers.^26–28^ For example, transgender and gender diverse Americans in a nationally representative study had nearly 3 times the rate of “problematic substance use” compared to cisgender respondents (31.2% vs 10.6%).^26^ While LGBTQIA+ communities consistently exhibit higher rates of substance use than heterosexual and/or cisgender communities, understanding whether certain LGBTQIA+ subcommunities (eg, cisgender gay men, bisexual cisgender women, transgender men) have markedly differential substance use rates compared to other LGBTQIA+ subcommunities may help target interventions and outreach efforts in an era of reduced federal funding for research and intervention with these communities.

Similar to experiences of IPV, structural stress derived from existing as an LGBTQIA+ person in a cisheternormative society is shown to increase the risk of substance use.^26,29–31^ In one study, exposure to stressors due to one’s sexual orientation and gender identity were associated with 15% and 39%, respectively, higher risk of problematic cannabis use compared to those without such exposures.^32^

Given the common theoretical frameworks linking IPV and substance use (eg, minority stress, intersectionality) within LGBTQIA+ communities, these 2 constructs are likely related. However, previous work in LGBTQIA+ communities has only focused on cross-sectional associations between IPV and substance use, and there are limited data to understand whether a temporal relationship exists. A better understanding of this relationship could promote integrating IPV-related content into existing, evidence-based substance use reduction interventions for LGBTQIA+ people. To this end, this study aims to (1) examine the prospective association between IPV and substance use among a diverse, community-based sample of LGBTQIA+ adults in the United States; (2) investigate whether different types (physical, sexual, and emotional) and frequency of IPV are associated with prospective substance use; and (3) identify potential disparities in these associations across gender groups within LGBTQIA+ communities. We hypothesize that (1) individuals with IPV exposure are more likely to use illicit substances in the subsequent year and report higher severity of substance use compared to their nonexposed counterparts, (2) all types of IPV will have positive associations with specific substances or substance use patterns, and (3) the relationship between IPV and substance use will differ based on gender groups.

## Methods

### Study design and participants

This study used data from The Population Research in Identity and Disparities for Equality (PRIDE) Study—a community-engaged, prospective cohort study of LGBTQIA+ adults in the United States. Details regarding the study design, recruitment, and retention are described elsewhere.^30,31,33^ For the present analysis, we used data from 2 annual questionnaires from The PRIDE Study: 2021 (collected July 2021-May 2022) and 2022 (collected June 2022-May 2023). Participants were included if they provided their gender identity and sexual orientation, experiences of IPV in the 2021 questionnaire, and patterns of substance use in the 2022 questionnaire. The PRIDE Study was approved by the Institutional Review Boards of the University of California, San Francisco, WIRB-Copernicus Group, and Stanford University.

### Measures

#### Extended-Hurt, Insulted, Threaten, Scream

The Extended-Hurt, Insulted, Threaten, Scream (E-HITS) scale is a 5-item screening tool that evaluates past-year IPV frequency.^34^ Participants are asked: “Over the last 12 months, how often did your partner: (1) physically hurt you?, (2) insult you or talk down to you?, (3) threaten you with harm?, (4) scream or curse at you?, and (5) force you to have sexual activities?” Responses are rated from 1 (Never) to 5 (Frequently), resulting in a total score ranging from 5 to 25. We classified the E-HITS items into 3 subtypes measuring physical (items 1 and 3), sexual (item 5), and emotional (items 2 and 4) past-year IPV.^35^ Summed scores for physical and emotional IPV had a total range from 2 to 10. We derived binary indicators for past-year IPV and each subtype. Unfortunately, there are no short-form IPV measures validated across LGBTQIA+ communities. However, the HITS screener (to which a single item was added to create E-HITS) has been validated in both cisgender women and men, making it one of the only short-form IPV screeners to perform well across genders.^36^ The HITS and E-HITS screeners have been used in a wide variety of contexts^34^ and language in the measures is broad (encompassing many potential forms of violence) and gender-neutral (adding to inclusivity across a wide variety of genders and sexual orientations in The PRIDE Study), making it an appropriate choice for this study. Similarly, while E-HITS subscales have not been formally validated, previous psychometric data from cisgender women found no statistical differences between the sensitivity and specificity of the E-HITS compared to the “gold standard” Conflict Tactics Scale-2 in detecting IPV overall or its subtypes.^34,35^ This provides preliminary justification for using the E-HITS to screen for IPV typology. We created a continuous (eg, frequency of IPV) and binary IPV exposures. For the binary exposure, participants who reported “Never” to all E-HITS items were categorized as not having experienced past-year IPV; all other participants were categorized as having experienced past-year IPV or one of its subtypes.

#### National Institute on Drug Abuse Modified Alcohol, Smoking and Substance Involvement Screening Test

The National Institute on Drug Abuse Modified Alcohol, Smoking and Substance Involvement Screening Test (NM ASSIST) is a screening tool that assesses the frequency of use, consequences of use, concerns about use from friends or relatives, and failure to stop or reduce use among 10 categories of illicit substances (listed below).^37^ In addition to measuring the presence of absence of use, the measure also includes assessments of “substance involvement”—the frequency, intensity, and consequences of use as well as behaviors associated with substance use that may indicate risk for a substance use disorder.

The PRIDE Study expanded the NM ASSIST to include additional substances not in the original tool based on community use patterns, and screen for all of the following substances: cannabis, cocaine, prescription stimulants (eg, “Ritalin” [methylphenidate]), methamphetamine, inhalants, inhaled nitrates (eg, poppers), sedatives (eg, “Xanax” [alprazolam], gamma-hydroxybutyric acid [GHB]), hallucinogens (eg, ecstasy, mushrooms), street opioids (eg, heroin), prescription opioids (eg, “Percocet” [oxycodone]), 3,4-methylenedioxy methamphetamine (MDMA, eg, “Ecstasy”), and “other.”

A binary outcome of any recent (past 30 days) substance use, and a continuous outcome of the NM ASSIST substance involvement scores were calculated as outcomes. Recent use was defined as reporting last use of at least 1 of the 13 possible substances in the survey within the past 30 days. Following standard scoring techniques,^38^ substance-specific involvement scores were calculated as the sum of responses from NM ASSIST items 2 to 7 for each substance, which included past 3-month questions on substance use frequency (item 2), urge to use (item 3), problems related to use (item 4), and interference with responsibilities (item 5), as well as lifetime questions on concern from friends and relatives about use (item 6) and failure to stop or reduce use (item 7). Participants responded to each item on a 5-point scale ranging from “Never” to “Daily or almost daily,” except for items 6 and 7 which are scores with values of 0 (No, never), 3 (Yes, but not in the past 3 months), and 6 (Yes, in the past 3 months). We then summed specific substance involvement scores into broader classes of substance use: stimulants (prescription stimulants, methamphetamine, and cocaine), hallucinogens (MDMA and other hallucinogens), opioids (prescription and street opioids), sedatives (GHB and other sedatives), and inhalants (poppers and other inhalants), similar to McNeely et al.^39^ Due to the different number of substances included in each class, the total scores for cannabis ranged from 0 to 39; from 0 to 117 for stimulants; and 0 to 78 each for hallucinogens, narcotics, sedatives, and inhalants. This helps us understand which classes may be most sensitive to change post-IPV.

#### History of substance use

We used 3 self-reported measures from the 2021 annual questionnaire to control for the history of substance use at baseline: lifetime substance use, past year substance use, and substance use disorder diagnosis. Participants are asked: “In your LIFETIME, which of the following substances have you ever used—either prescribed or not prescribed by a health care provider?” Those who selected at least 1 substance were categorized as having any lifetime use. Past year substance use was defined as reporting last use of at least 1 of the 13 possible substances within the past 12 months. Diagnosis of substance use disorder was identified by the question: “Do you currently have any of the following conditions that have been diagnosed by a health care provider?” Participants who selected “Drug or Substance Use Disorder” were categorized as having received a diagnosis for substance use disorder. Given emerging evidence suggesting that current substance use can mediate future use,^40,41^ we chose to use lifetime scores rather than 2021 substance use to allow for this potential mediating effect.

#### Relationship characteristics

The 2021 annual questionnaire collected information on participants’ relationship status and current relationship satisfaction. Participants are asked: “Are you currently in a relationship?” Those who responded “yes” were asked about their current romantic relationship(s): “In general, how satisfied are you with your current romantic relationship(s)?” Responses, ranged from 0 (Very dissatisfied) to 4 (Very satisfied).

#### Sociodemographics

Participants self-reported their gender identity, sex assigned at birth, sexual orientation, ethnoracial identity, education level, and annual individual income. The option to select multiple responses was provided for gender identity, sexual orientation, and ethnoracial identity. To further describe the sample, we categorized participants into gender groups (cisgender man, cisgender woman, gender diverse assigned female at birth [AFAB], gender diverse assigned male at birth [AMAB], transgender man, and transgender woman) using the 2-step approach,^42,43^ and sexual orientation groups (asexual, bisexual, gay or lesbian, pansexual, queer, heterosexual, multiple options selected, other). We calculated age and census region from self-reported birth date and ZIP code.

### Statistical analyses

Modified Poisson regression models were used to evaluate associations between past-year IPV (and its subtypes) and recent substance use. Linear regression models were used to assess associations between past-year IPV and each substance-specific NM ASSIST score. Unadjusted and fully adjusted models controlling for age, gender group, sexual orientation group, education, employment, income, census region, lifetime substance use, and substance use disorder diagnosis were fit for each exposure-outcome pair. Robust standard errors for all models were estimated using the Huber-White sandwich estimator to address potential violations of model-based assumptions involving the variance of the outcome.^44^ Missingness was handled using multiple imputations via chained equations,^45^ generating 20 imputed datasets under the assumption of missing at random.

We conducted 5 supplementary analyses. First, we restricted the sample to those in a current relationship as they might experience increased vulnerability to IPV and with greater severity^46,47^ and also adjusted for relationship satisfaction. Second, we stratified models by gender groups given that IPV experiences may differ by gender group.^48,49^ Third, we re-fitted our models using each substance class’s maximum and mean substance involvement score. Scores for each substance class using the maximum approach range from 0 to 39, with higher scores indicating greater substance involvement. Fourth, we adjusted for past year rather than lifetime substance use. Last, we explored a reciprocal association between past 30-day substance use (2021) and past-year IPV (2022). Estimates from Poisson models were exponentiated to derive risk ratios (RR). Statistical significance was considered as a 95% confidence interval (CI) excluding 0 for linear models and 1 for Poisson models, assuming a type 1 error rate of 0.05 (2-sided) using R version 4.2.1.^50^

## Results

The analytic sample included 3745 participants with a median age of 34.3 years (interquartile range: 27.6–48.5; Table 1). About half (49.2%) identified as transgender or gender diverse. Nearly half (47.0%) identified with multiple sexual orientations. Most participants identified only as White (82.2%) with an additional 10.5% reporting White in addition to another ethnoracial identity. One-third (33.1%, *n* = 1240) resided in the Pacific division, where the study is based. Two-thirds (66.0%, *n* = 2472) reported currently being in a relationship, 56.8% of whom (*n* = 1557) reported being satisfied or very satisfied with their current relationship. About 85.5% (*n* = 3202) reported any lifetime substance use, and 2.3% (*n* = 86) reported receiving a diagnosis of a substance use disorder.

At baseline in 2021, about one-quarter (24.6%, *n* = 921) of participants reported any exposure to past-year IPV with a mean E-HITS score of 5.65 (standard deviation [SD]: 1.60; Table 2). Among subtypes, 3.5% (*n* = 131) reported any physical IPV (mean: 2.06; SD: 0.42), 2.3% (*n* = 86) reported any sexual IPV (mean: 1.04; SD: 0.29), and 23.8% (*n* = 891) reported any emotional IPV (mean: 2.54; SD: 1.24). In the adjusted model, any past-year IPV (aRR: 1.11; 95% CI, 1.03–1.19), physical IPV (aRR: 1.25; 95% CI, 1.10–1.42), and emotional IPV (aRR: 1.11; 95% CI, 1.03–1.19) remained associated with recent substance use. No association was observed between past-year sexual IPV and recent substance use.

Substance involvement scores varied across different substances with participants reporting the highest score for cannabis (mean: 3.46; SD: 5.73) and the lowest score for inhalants (mean: 0.49; SD: 2.03; Table 3). In the unadjusted model, exposure to IPV in 2021 was associated with a significantly higher substance involvement score for every substance type in 2022. After adjustment, past-year (2021) IPV was associated with higher 2022 scores for cannabis (*B*: 1.03; 95% CI, 0.57–1.50), stimulants (*B*: 0.48; 95% CI, 0.11–0.84), hallucinogens (*B*: 0.24; 95% CI, 0.07–0.41), and narcotics (*B*: 0.27; 95% CI, 0.06–0.49). Results were similar when examining the associations between IPV frequency in 2021 (continuous E-HITS scores) and 2022 substance involvement scores (Table 4). In the adjusted model, a higher E-HITS score was associated with a higher score for cannabis (*B*: 0.22; 95% CI, 0.08–0.36), stimulants (*B*: 0.20; 95% CI, 0.06–0.35), hallucinogens (*B*: 0.07; 95% CI, 0.02–0.12), and narcotics (*B*: 0.12; 95% CI, 0.03–0.20). Full model outputs are presented in Tables S1–S3.

After restricting the sample to participants who were in relationships and adjusting for relationship satisfaction, associations between 2021 exposure to IPV, its subtypes, and IPV severity with substance use and substance involvement scores in 2022 were consistent with the main analyses (Tables S4–S7). Although associations between any past-year IPV and past 30-day substance use did not differ by gender groups (Table S8), there were variations in the associations between past-year IPV and substance involvement scores (Table S9). Exposure to past-year (2021) IPV was associated with higher scores for stimulants (*B*: 1.48; 95% CI, 0.44–2.52) and opiates (*B*: 0.49; 95% CI, 0.05–0.94) in 2022 among cisgender sexual minority men, higher scores for cannabis (*B*: 1.17; 95% CI, 0.41–1.92) and opiates (*B*: 0.44; 95% CI, 0.10–0.78) among cisgender sexual minority women, higher scores for sedatives among gender diverse people AFAB (*B*: 0.46; 95% CI, 0.01–0.92), and higher scores for inhalants among gender diverse people AMAB (*B*: 0.79; 95% CI, 0.05–1.52). Results using maximum NM ASSIST scores for each substance class yielded similar results to the summed NM ASSIST scores, while results using mean NM ASSIST score were similar but slightly attenuated (Tables S10 and S11). Adjustment of past year rather than lifetime substance use resulted in slightly attenuated associations (Tables S12 and S13). Finally, reversing the temporal order showed a reciprocal association between past 30-day substance use in 2021 and past-year IPV in 2022 (RR: 1.28; 95% CI, 1.13–1.44).

## Discussion

While it is well known that IPV often occurs in the context of increased substance use,^28,32^ our results provide some of the first data on how IPV may itself lead to increased substance use in this population. This may lead to insights into how IPV may influence these types of stress-response behaviors in the LGBTQIA+ community writ large and within subcommunities. Data indicated a significant burden of IPV with one-quarter (24.6%) of respondents experiencing relationship violence in the past 12 months. More participants endorsed experiencing emotional violence (23.8%) compared to physical (3.5%) and sexual (2.3%). This mirrors other studies of LGBTQIA+ adults who endorsed higher rates of experiencing emotional or psychological IPV compared to physical or sexual IPV.^7,51^ Average scores for all drug classes fell into NIDA’s “lower risk” category.

Those who reported experiencing any type of IPV had significantly higher scores and higher risk for past 30-day use for all drug classes than those who did not endorse IPV. While potential causal pathways were not assessed in this study, the results lend credence to the overall stress-response model of understanding IPV in LGBTQIA+ people and are consistent with the results of our companion study on IPV and alcohol use.^52^ While we have framed this analysis around stressors at the structural level, additional theories of stress and coping^53,54^ (eg, Family Stress Theory,^55^ General Strain Theory,^56^ Lifecourse Theory^57^) may also help explain how the stress and trauma of experiencing IPV can lead to substance use in LGBTQIA+ communities. The specific ways in which IPV influences substance use remain unclear in LGBTQIA+ communities, and as this study and the companion paper show, also differ significantly by the substance studied and nature of IPV experienced. In the companion study, we modeled quadratic equations and note a curvilinear relationship between IPV and alcohol such that the marginal increase in alcohol use decreased as IPV frequency increased.^52^ While the multiple substances examined in the current study preclude the inclusion of twice the number of models and results, this suggests that IPV should not be treated as a binary variable and should be assessed over time. In particular, longitudinal research with LGBTQIA+ people experiencing IPV is needed to begin identifying specific causal pathways and more granular relationships between IPV and both alcohol and substance use. Additionally, such research could help parse how to design and improve trauma-informed secondary prevention interventions to reduce IPV survivors’ future substance use. We posit that including screening for IPV and referral to LGBTQIA+ affirming services may help reduce future substance use.

The E-HITS screener calculates a continuous measure of IPV frequency. In this study, each additional point on the E-HITS screener was associated with a significant increase in substance involvement scores for all substance classes. The largest effect was for cannabis, with each E-HITS point contributing to an increase of 0.22 points on the NM ASSIST scale. While not specifically assessed in this study, such a linear relationship could point to the accumulation of stress over time in the context of chronic violence exposure.^58^ As The PRIDE Study continues, we aim to continue expanding our understanding of how the frequency of IPV contributes to substance use over time, allowing for an improved understanding of how IPV contributes to the broader understanding of the impacts of (intersectional) minority stress on the health of LGBTQIA+ people.

Specific to cannabis use, results indicated that those who experience any type of IPV had an average substance involvement score of 4.12 (SD: 6.13) compared to 3.20 (SD: 5.27) for those who did not experience IPV. Considering NM ASSIST scoring suggests 0–3 (only whole numbers are used in individual assessments) be used as the range for “lower risk,”^37^ the treatment protocol for a person with a score of 4.0 is different from someone with a score of 3.0. For those with a substance involvement score greater or equal to 4, “brief intervention” (ie, the traditional 3- to 15-minute motivational interviewing intervention recommended by most screening, brief intervention, and referral to treatment protocols) is recommended, while “brief education” (ie, a less formal conversation informing patients about the risks of illicit drug use and signs of a substance use disorder) is recommended for those with scores between 0 and 3.^59^ This means that IPV exposure is one factor that can alter the clinical approach to substance use education and treatment in LGBTQIA+ people. Unfortunately, screening for IPV among LGBTQIA+ people remains rare in clinical settings, and no IPV screening tools are validated in LGBTQIA+ communities.^60^ Similarly, substance use brief interventions may be improved if they integrated discussions of IPV and provide relevant resources and referrals for survivors.

Results that physical and emotional IPV were significantly associated with increased substance use, while sexual violence was not may seem counter intuitive. However, one reason for this null finding may be the way sexual IPV was measured. The single-item question regarding sexual violence, while broad, refers only to “sexual activities.” Types of sexual IPV such as sexual coercion, revoked consent, and online forms of sexual IPV may not be captured by this item. Given the small number of respondents who endorsed sexual IPV at baseline (2.3%), we are likely underpowered to detect a relationship between sexual IPV and recent substance use.

The E-HITS does not cover all types of IPV behaviors experienced by LGBTQIA+ individuals. For example, digital stalking (eg, via forced location sharing) and other forms of technology-facilitated violence are not captured in E-HITS, but they are relevant queer communities.^61^ Since E-HITS was not validated in LGBTQIA+ samples, we used more sensitive binary and continuous measures of violence instead of the traditional HITS cutoff scores suggested by Sherin et al^62^ (≥10) or Iverson et al^63^ (≥7). It is critical that additional work be conducted to develop more nuanced and rigorous population-specific measures of IPV that can help better illuminate how different IPV typologies affect substance use in this population.^64^

Research examining IPV within LGBTQIA+ communities tends to focus on individuals currently in relationships,^65,66^ potentially overlooking many who have experienced IPV but who might have recently left a relationship due to IPV or not report as having been in a relationship at all. We suggest that relationship status should not determine inclusion in studies examining this topic. This recommendation arises from our analysis of those currently in relationships, accounting for relationship status and satisfaction, which did not find significant differences from findings from the entire sample. Consequently, interventions aimed at educating about relationship dynamics (eg, choice of partners, better communication, etc) could reduce their odds of entering abusive relationships.

Finally, LGBTQIA+ people form a diverse, intersectional community with overlapping and sometimes fluid sexual orientations and gender identities that can significantly alter their risk for IPV.^9^ While exploratory, this study aimed to understand the broad effects of IPV on substance use in this community and important nuances emerged from the sensitivity analyses by gender groups. Although the association between IPV and frequency of substance use (as measured by past 30-day use) did not differ by group, the types of substance use associated with IPV exposure did. For example, reporting IPV was significantly associated with reporting prescription stimulant use among cisgender men. Given the current resurgence of stimulant use among gay and bisexual men,^67,68^ additional inquiry into this relationship is prudent. Among cisgender women, however, IPV exposure was most significantly related to increased cannabis use, an important finding considering lesbian and bisexual women use cannabis at more than twice the rate of the general population. Among gender minority groups, IPV was associated with increased sedative (gender diverse AFAB) and inhalant (gender diverse AMAB) use. Sedative and inhalant use are especially concerning given the increasing number of deaths associated with use of recreational GHB (a sedative) and amyl nitrate (an inhalant).^69–71^

While the exact role of IPV in specific substance choices remains unclear, the results of this analysis challenge researchers and clinicians to more consistently and meaningfully include experiences of IPV in their conceptual models and frameworks for substance use disparities in LGBTQIA+ populations. The inclusion of IPV- and of trauma exposure as a key driver of substance use can contribute our understanding of these disparities. Specifically, future work should further examine why survivors of IPV from LGBTQIA+ subcommunities and with different experiences of minority stress turn to specific substances, and whether interventions such as social support or trauma recovery services can moderate the relationship between IPV and substance use. Further, researchers, clinicians, and community members should advocate for holistic substance use prevention interventions that incorporate healthy relationships content to prevent IPV before it can lead to increased substance use.

### Limitations

There are several limitations to our analyses. First, the E-HITS scale is an IPV screener and is not validated in, nor able to capture the full experience of IPV among, LGBTQIA+ individuals. As previously noted, there are important forms of violence that may be particularly salient for LGBTQIA+ people but fall outside the traditional IPV measures and remain unaccounted in most IPV studies given the lack of validated short-form measures. Second, we did not measure polydrug use, or the concurrent use of more than 1 substance at a time, which is increasingly common across communities.^72^ Future studies examine how a constellation of substance use is related to IPV. Third, while The PRIDE Study is a diverse sample in many ways, racial and ethnic minority communities are underrepresented. This means The PRIDE Study may not be generalizable to all LGBTQIA+ subgroups, especially those that identify as belonging to an ethnic or racial minority group. Fourth, given that E-HITS was only included in The PRIDE Study beginning in 2021, we could only examine the 2 most recent years of the study. Considering the recall period for this measure included 2020 and 2021, the COVID-19 pandemic may have affected rates of IPV and substance use since both increased during the height of the pandemic.^9^ Finally, E-HITS only screens for IPV victimization, but mounting evidence suggests that LGBTQIA+ relationships have high rates of bidirectional violence.^73^ We may, therefore, be missing the full spectrum of violence occurring in these relationships. As The PRIDE Study continues, we will draw longer-term inferences on the effects of IPV on substance use.

## Conclusion

This study provides preliminary evidence for the temporal relationship between IPV and substance use in a large, diverse sample of LGBTQIA+ adults across the United States. Results corroborate previous findings that LGBTQIA+ people are deeply affected by relationship violence, which may lead to increases in substance use. By adding rigor to the existing evidence base, this study contributes to our long-term goal of developing individual, dyadic, and community-based interventions to interrupt the interconnected pathways of stress, violence, and substance use in LGBTQIA+ communities. However, the development of such interventions requires better IPV measures, increased affirming community resources for LGBTQIA+ survivors, and more research into the causal pathways inherent in the relationships mentioned above—are sorely needed. While preliminary, this study strengthens the foundation for research, advocacy, and policy necessary to achieve health equity for LGBTQIA+ communities.

## Supplementary Material

Supplementary Materials

Supplementary material is available at *Annals of Behavioral Medicine* online.

## Figures and Tables

**Table 1.** Sociodemographics, relationship characteristics, and substance use history among sexual and gender minority participants stratified by past-year IPV (E-HITS).

	Any past year IPV		
	Total	No	Yes
	(*n* = 3745)	(*n* = 2823)	(*n* = 922)
**Age, years (median, IQR)**	34.3 (27.6–48.5)	33.7 (27.2–47.2)	36.8 (29.0–51.1)
**Gender identity^a^ (*n*, %)**			
**Agender**	206 (5.5)	170 (6.0)	36 (3.9)
**Cisgender man**	639 (17.1)	465 (16.5)	174 (18.9)
**Cisgender woman**	878 (23.4)	665 (23.6)	213 (23.1)
**Genderqueer**	541 (14.4)	405 (14.3)	136 (14.8)
**Man**	778 (20.8)	595 (21.1)	183 (19.8)
**Nonbinary**	975 (26.0)	754 (26.7)	221 (24.0)
**Questioning**	177 (4.7)	138 (4.9)	39 (4.2)
**Transgender man**	516 (13.8)	389 (13.8)	127 (13.8)
**Transgender woman**	213 (5.7)	160 (5.7)	53 (5.7)
**Two-spirit**	41 (1.1)	30 (1.1)	11 (1.2)
**Woman**	801 (21.4)	586 (20.8)	215 (23.3)
**Another gender identity**	258 (6.9)	187 (6.6)	71 (7.7)
**Gender groups (*n*, %)**			
**Cisgender man**	921 (24.6)	683 (24.2)	238 (25.8)
**Cisgender woman**	982 (26.2)	730 (25.9)	252 (27.3)
**Gender diverse AFAB**	943 (25.2)	737 (26.1)	206 (22.3)
**Gender diverse AMAB**	138 (3.7)	99 (3.5)	39 (4.2)
**Transgender man**	539 (14.4)	409 (14.5)	130 (14.1)
**Transgender woman**	222 (5.9)	165 (5.8)	57 (6.2)
**Sexual orientation^a^ (*n*, %)**			
**Asexual**	435 (11.6)	369 (13.1)	66 (7.2)
**Bisexual**	1135 (30.3)	857 (30.4)	278 (30.2)
**Gay**	1278 (34.1)	956 (33.9)	322 (34.9)
**Lesbian**	832 (22.2)	603 (21.4)	229 (24.8)
**Pansexual**	591 (15.8)	411 (14.6)	180 (19.5)
**Queer**	1723 (46.0)	1307 (46.3)	416 (45.1)
**Questioning**	86 (2.3)	71 (2.5)	15 (1.6)
**Same-gender loving**	167 (4.5)	120 (4.3)	47 (5.1)
**Heterosexual**	72 (1.9)	48 (1.7)	24 (2.6)
**Two-spirit**	30 (0.8)	20 (0.7)	10 (1.1)
**Another sexual orientation**	162 (4.3)	123 (4.4)	39 (4.2)
**Sexual orientation groups (*n*, %)**			
**Asexual**	107 (2.9)	100 (3.5)	7 (0.8)
**Bisexual**	302 (8.1)	234 (8.3)	68 (7.4)
**Gay or Lesbian**	1115 (29.8)	823 (29.2)	292 (31.7)
**Pansexual**	94 (2.5)	64 (2.3)	30 (3.3)
**Queer**	307 (8.2)	239 (8.5)	68 (7.4)
**Heterosexual**	35 (0.9)	22 (0.8)	13 (1.4)
**Multiple options selected**	1762 (47.0)	1323 (46.9)	439 (47.6)
**Questioning, same-gender loving, or another sexual orientation**	23 (0.6)	18 (0.6)	5 (0.5)
**Ethnoracial identity^a,b^ (*n*, %)**			
**American Indian or Alaska Native**	106 (2.8)	74 (2.6)	32 (3.5)
**Asian**	171 (4.6)	145 (5.1)	26 (2.8)
**Black, African American or African**	136 (3.6)	106 (3.8)	30 (3.3)
**Hispanic, Latino or Spanish**	225 (6.0)	169 (6.0)	56 (6.1)
**Middle Eastern or North African**	54 (1.4)	40 (1.4)	14 (1.5)
**Native Hawaiian or other Pacific Islander**	8 (0.2)	8 (0.3)	0 (0.0)
**White**	3448 (92.1)	2581 (91.4)	867 (94.0)
**Another ethnoracial identity**	57 (1.5)	42 (1.5)	15 (1.6)
**Education level (*n*, %)**			
**High school or less**	142 (3.8)	99 (3.5)	43 (4.7)
**Some college**	695 (18.6)	505 (17.9)	190 (20.6)
**4-year college grad**	1309 (35.0)	1015 (36.0)	294 (31.9)
**Advanced degree**	1598 (42.7)	1203 (42.6)	395 (42.8)
**Missing**	1 (0.0)	1 (0.0)	0 (0.0)
**Current employment (*n*, %)**			
**No**	996 (26.6)	734 (26.0)	262 (28.4)
**Yes**	2747 (73.4)	2087 (73.9)	660 (71.6)
**Missing**	2 (0.1)	2 (0.1)	0 (0.0)
**Individual income (*n*, %)**			
**$0–20 000**	1080 (28.8)	821 (29.1)	259 (28.1)
**$20 001–50 000**	1101 (29.4)	844 (29.9)	257 (27.9)
**$50 001–100 000**	982 (26.2)	745 (26.4)	237 (25.7)
**$100 001+**	556 (14.8)	394 (14.0)	162 (17.6)
**Missing**	26 (0.7)	19 (0.7)	7 (0.8)
**Current census region (*n*, %)**			
**Northeast**	766 (20.5)	584 (20.7)	182 (19.7)
**Midwest**	764 (20.4)	571 (20.2)	193 (20.9)
**South**	951 (25.4)	718 (25.4)	233 (25.3)
**West**	1241 (33.1)	933 (33.0)	308 (33.4)
**Missing**	23 (0.6)	17 (0.6)	6 (0.7)
**Currently in relationship (*n*, %)**			
**No**	1250 (33.4)	1107 (39.2)	143 (15.5)
**Yes**	2470 (66.0)	1699 (60.2)	771 (83.6)
**Missing**	25 (0.7)	17 (0.6)	8 (0.9)
**Relationship satisfaction (*n*, %)**			
**Very dissatisfied**	49 (1.3)	17 (0.6)	32 (3.5)
**Dissatisfied**	110 (2.9)	42 (1.5)	68 (7.4)
**Neutral**	186 (5.0)	96 (3.4)	90 (9.8)
**Satisfied**	845 (22.6)	521 (18.5)	324 (35.1)
**Very satisfied**	1279 (34.2)	1022 (36.2)	257 (27.9)
**Missing**	1276 (34.1)	1125 (39.9)	151 (16.4)
**Lifetime substance use (*n*, %)**			
**No**	470 (12.6)	406 (14.4)	64 (6.9)
**Yes**	3203 (85.5)	2368 (83.9)	835 (90.6)
**Missing**	72 (1.9)	49 (1.7)	23 (2.5)
**Past-year substance use (*n*, %)**			
**No**	1381 (36.9)	1129 (40.0)	252 (27.3)
**Yes**	2292 (61.2)	1645 (58.3)	647 (70.2)
**Missing**	72 (1.9)	49 (1.7)	23 (2.5)
**Substance use disorder diagnosis (*n*, %)**	87 (2.3)	51 (1.8)	36 (3.9)

Abbreviations: IQR, interquartile range; AFAB, assigned female at birth; AMAB, assigned male at birth; E-HITS, Extended-Hurt, Insulted, Threaten, Scream.

a Participants could select multiple responses; thus, the sum of percentages is greater than 100%.

b About 10.5% selected multiple ethnoracial identities.

**Table 2.** Association between intimate partner violence and its subtypes with past 30-day substance use among sexual and gender minority participants in The PRIDE Study, 2021–2022.^a^

Exposure type	Exposure	Total		Past 30-day substance use	Unadjusted RR(95% CI)	Adjusted RR(95% CI)
		(*n* = 3745)		(*n* = 1803)		
		Mean (SD)	*n* (%)	*n* (Row %)		
**Overall**	E-HITS	5.65 (1.60)				
	No		2823 (75.4)	1295 (45.9)	Ref	Ref
	Yes		922 (24.6)	508 (55.1)	**1.20 (1.12–1.29)**	**1.11 (1.03–1.19)**
**Type of E-HITS**	Physical	2.06 (0.42)				
	No		3614 (96.5)	1714 (47.4)	Ref	Ref
	Yes		131 (3.5)	89 (67.9)	**1.43 (1.27–1.62)**	**1.25 (1.10–1.42)**
	Sexual	1.04 (0.29)				
	No		3659 (97.7)	1756 (48.0)	Ref	Ref
	Yes		86 (2.3)	47 (54.7)	1.14 (0.94–1.38)	0.95 (0.77–1.16)
	Emotional	2.54 (1.24)				
	No		2854 (76.2)	1311 (45.9)	Ref	Ref
	Yes		891 (23.8)	492 (55.2)	**1.20 (1.12–1.29)**	**1.11 (1.04–1.20)**

Abbreviations: E-HITS, Extended-Hurt, Insulted, Threaten, Scream; RR, risk ratio; CI, confidence interval.

a Models adjusted for age (continuous), gender identity, sexual orientation, education, employment, income, census region, lifetime substance use, and substance use disorder. Bolded estimates indicate *P* < .05.

**Table 3.** Association between any past-year intimate partner violence (2021) and NM ASSIST substance involvement scores (2022) for specific drug class among sexual and gender minority participants in The PRIDE Study.^a^

ASSIST scores	Total	E-HITS		Unadjusted *B* (95% CI)	Adjusted *B* (95% CI)
		No	Yes		
	(*n* = 3745)	(*n* = 2823)	(*n* = 922)		
	Mean (SD)	Mean (SD)	Mean (SD)		
**Cannabis**	3.46 (5.73)	3.18 (5.50)	4.32 (6.30)	**1.15 (0.69–1.60)**	**1.03 (0.57–1.50)**
**Stimulants**	1.68 (4.47)	1.46 (3.88)	2.38 (5.89)	**0.92 (0.51–1.33)**	**0.48 (0.11–0.84)**
**Hallucinogens**	0.50 (1.96)	0.41 (1.70)	0.74 (2.57)	**0.33 (0.15–0.51)**	**0.24 (0.07–0.41)**
**Opiates**	0.61 (2.39)	0.50 (2.11)	0.95 (3.07)	**0.45 (0.24–0.66)**	**0.27 (0.06–0.49)**
**Sedatives**	1.13 (3.04)	1.00 (2.88)	1.56 (3.44)	**0.57 (0.32–0.81)**	0.23 (−0.02 to 0.49)
**Inhalants**	0.49 (2.03)	0.43 (1.84)	0.68 (2.52)	**0.25 (0.08–0.43)**	0.17 (−0.01 to 0.35)

Abbreviations: NM ASSIST, National Institute of Drug Abuse Modified Alcohol, Smoking and Substance Involvement Screening Test; E-HITS, Extended-Hurt, Insulted, Threaten, Scream; CI, confidence interval.

a Models adjusted for age (continuous), gender identity, sexual orientation, education, employment, income, census region, lifetime substance use, and substance use disorder. Bolded estimates indicate *P* < .05.

**Table 4.** Association between intimate partner violence frequency (E-HITS scores, 2021) and NM ASSIST substance involvement scores (2022) for specific drug class among sexual and gender minority participants in The PRIDE Study.^a^

ASSIST Scores	Unadjusted *B*	Adjusted *B*
	(95% CI)	(95% CI)
**Cannabis**	**0.28 (0.15–0.41)**	**0.22 (0.08–0.36)**
**Stimulants**	**0.34 (0.19–0.49)**	**0.20 (0.06–0.35)**
**Hallucinogens**	**0.09 (0.04–0.14)**	**0.07 (0.02–0.12)**
**Opiates**	**0.17 (0.09–0.24)**	**0.12 (0.03–0.20)**
**Sedatives**	**0.14 (0.06–0.21)**	0.04 (−0.04 to 0.12)
**Inhalants**	**0.07 (0.02–0.12)**	0.03 (−0.01 to 0.10)

Abbreviations: NM ASSIST, National Institute of Drug Abuse Modified Alcohol, Smoking and Substance Involvement Screening Test; E-HITS, Extended-Hurt, Insulted, Threaten, Scream; CI, confidence interval.

a Models adjusted for age (continuous), gender identity, sexual orientation, education, employment, income, census region, lifetime substance use, and substance use disorder. Overall E-HITS scores were mean centered. Bolded estimates indicate *P* < .05.

## Data Availability

Data underlying the study cannot be made publicly available due to ethical concerns about participant confidentiality. Researchers interested in The PRIDE Study data may submit a brief application which is reviewed by a Research Advisory Committee (composed of scientists) and Participant Advisory Committee (composed of participants) to affirm appropriate data use. Details about the Ancillary Study process are available at www.pridestudy.org/collaborate or by contacting support@pridestudy.org or 855–421-9991 (toll-free).
