# Supplementary material for: Exposure to intimate partner violence and subsequent substance use among a nationwide sample of LGBTQIA+ people: results of The PRIDE Study
Source: Ann Behav Med. Author manuscript; Available in PMC 2025 Dec 22. (PMC12602189; doi:10.1093/abm/kaaf091)
Supplement: Supplementary Materials [file NIHMS2122614-supplement-Supplementary_Materials.docx]

## **Supplemental Materials**

[**Supplemental Table 1.** Full model output of the association between intimate partner violence and its subtypes with past 30-day substance use among sexual and gender minority participants in The PRIDE Study, 2021-2022 2](#_Toc197603825)

[**Supplemental Table 2.** Full model output of the association between any past-year intimate partner violence (2021) and NM ASSIST substance involvement scores (2022) for specific drug class among sexual and gender minority participants in The PRIDE Study 4](#_Toc197603826)

[**Supplemental Table 3.** Full model output of the association between intimate partner violence frequency (E-HITS scores, 2021) and NM ASSIST substance involvement scores (2022) for specific drug class among sexual and gender minority participants in The PRIDE Study 7](#_Toc197603827)

[**Supplemental Table 4.** Participants characteristics by current relationship status 10](#_Toc197603828)

[**Supplemental Table 5.** Association between intimate partner violence and its subtypes with any past 30-day substance use among sexual and gender minority participants who are currently in a relationship in The PRIDE Study, 2021-2022 12](#_Toc197603829)

[**Supplemental Table 6.** Association between any past-year intimate partner violence and NM ASSIST substance involvement scores for specific drug class among sexual and gender minority participants who are currently in a relationship in The PRIDE Study, 2021-2022 13](#_Toc197603830)

[**Supplemental Table 7.** Association between intimate partner violence severity (E-HITS scores) and NM ASSIST substance involvement scores for specific drug class among sexual and gender minority participants who are currently in a relationship in The PRIDE Study, 2021-2022 14](#_Toc197603831)

[**Supplemental Table 8.** Association between intimate partner violence and past 30-day substance use by gender groups among sexual and gender minority participants in The PRIDE Study, 2021-2022 15](#_Toc197603832)

[**Supplemental Table 9.** Association between past-year intimate partner violence and NM ASSIST substance involvement scores for specific drug class by gender groups among sexual and gender minority participants in The PRIDE Study, 2021-2022 16](#_Toc197603833)

[**Supplemental Table 10.** Association between past-year intimate partner violence (2021) and NM ASSIST substance involvement scores (2022), using the maximum and mean substance involvement score within each drug class, among sexual and gender minority participants in The PRIDE Study 17](#_Toc197603834)

[**Supplemental Table 11.** Association between intimate partner violence frequency (E-HITS scores, 2021) and NM ASSIST substance involvement scores (2022), using the maximum and mean substance involvement score within each drug class, among sexual and gender minority participants in The PRIDE Study 18](#_Toc197603835)

[**Supplemental Table 12.** Association between intimate partner violence and its subtypes (2021) with past 30-day substance use (2022) adjusting for past year substance use in 2021 among sexual and gender minority participants in The PRIDE Study, 2021-2022 19](#_Toc197603836)

[**Supplemental Table 13.** Association between intimate partner violence (2021) and NM ASSIST substance involvement scores (2022) adjusting for past year substance use in 2021 among sexual and gender minority participants in The PRIDE Study, 2021-2022 20](#_Toc197603837)

**Supplemental Table 1.** Full model output of the association between intimate partner violence and its subtypes with past 30-day substance use among sexual and gender minority participants in The PRIDE Study, 2021-2022

|  |  | **Overall** | | | | **Physical** | | | | **Sexual** | | | | **Emotional** | | | |
| --- | --- | --- | --- | --- | --- | --- | --- | --- | --- | --- | --- | --- | --- | --- | --- | --- | --- |
|  |  | aRR | | 95% CI | | aRR | | 95% CI | | aRR | | 95% CI | | aRR | | 95% CI | |
| E-HITS (ref = No) | | 1.11 | 1.03 | | 1.19 | 1.25 | 1.10 | | 1.42 | 0.95 | 0.77 | | 1.16 | 1.11 | 1.04 | | 1.20 |
| Age, years | | 0.99 | 0.99 | | 1.00 | 0.99 | 0.99 | | 1.00 | 0.99 | 0.99 | | 1.00 | 0.99 | 0.99 | | 1.00 |
| Gender groups (ref = Cisgender man) | |  |  | |  |  |  | |  |  |  | |  |  |  | |  |
|  | Cisgender woman | 0.9 | 0.77 | | 1.05 | 0.90 | 0.77 | | 1.05 | 0.90 | 0.77 | | 1.05 | 0.90 | 0.77 | | 1.05 |
|  | Gender diverse AFAB | 1.09 | 0.93 | | 1.29 | 1.09 | 0.92 | | 1.28 | 1.09 | 0.92 | | 1.28 | 1.09 | 0.92 | | 1.28 |
|  | Gender diverse AMAB | 1.08 | 0.82 | | 1.41 | 1.07 | 0.82 | | 1.40 | 1.08 | 0.83 | | 1.42 | 1.08 | 0.82 | | 1.41 |
|  | Transgender man | 1.11 | 0.93 | | 1.33 | 1.11 | 0.92 | | 1.33 | 1.10 | 0.92 | | 1.32 | 1.11 | 0.92 | | 1.33 |
|  | Transgender woman | 1.06 | 0.84 | | 1.33 | 1.05 | 0.84 | | 1.32 | 1.06 | 0.84 | | 1.33 | 1.05 | 0.84 | | 1.33 |
| Sexual orientation groups (ref = Gay or Lesbian) | |  |  | |  |  |  | |  |  |  | |  |  |  | |  |
|  | Asexual | 0.68 | 0.45 | | 1.04 | 0.68 | 0.45 | | 1.02 | 0.67 | 0.44 | | 1.01 | 0.68 | 0.45 | | 1.03 |
|  | Bisexual | 1.04 | 0.86 | | 1.27 | 1.04 | 0.85 | | 1.27 | 1.04 | 0.86 | | 1.27 | 1.04 | 0.86 | | 1.27 |
|  | Pansexual | 1.18 | 0.88 | | 1.59 | 1.20 | 0.89 | | 1.60 | 1.20 | 0.90 | | 1.61 | 1.18 | 0.88 | | 1.59 |
|  | Queer | 1.05 | 0.86 | | 1.29 | 1.05 | 0.86 | | 1.29 | 1.05 | 0.86 | | 1.29 | 1.05 | 0.86 | | 1.29 |
|  | Heterosexual | 0.91 | 0.54 | | 1.52 | 0.92 | 0.55 | | 1.54 | 0.92 | 0.55 | | 1.55 | 0.91 | 0.54 | | 1.52 |
|  | Multiple options selected | 1.03 | 0.9 | | 1.18 | 1.03 | 0.90 | | 1.18 | 1.03 | 0.90 | | 1.18 | 1.03 | 0.90 | | 1.18 |
|  | Questioning, same-gender loving, or another sexual orientation | 0.53 | 0.23 | | 1.19 | 0.53 | 0.24 | | 1.21 | 0.53 | 0.23 | | 1.19 | 0.53 | 0.24 | | 1.20 |
| Education level (ref = HS or less) | |  |  | |  |  |  | |  |  |  | |  |  |  | |  |
|  | Some college | 1.15 | 0.88 | | 1.51 | 1.14 | 0.87 | | 1.49 | 1.14 | 0.87 | | 1.49 | 1.15 | 0.88 | | 1.51 |
|  | 4-year college grad | 1.08 | 0.83 | | 1.41 | 1.07 | 0.82 | | 1.40 | 1.06 | 0.82 | | 1.39 | 1.09 | 0.83 | | 1.42 |
|  | Advanced degree | 1.00 | 0.77 | | 1.32 | 0.99 | 0.75 | | 1.30 | 0.99 | 0.75 | | 1.29 | 1.01 | 0.77 | | 1.32 |
| Current Employment (ref = No) | | 0.98 | 0.86 | | 1.1 | 0.98 | 0.87 | | 1.10 | 0.97 | 0.86 | | 1.10 | 0.98 | 0.86 | | 1.10 |
| Individual Income (ref = $0-20,000) | |  |  | |  |  |  | |  |  |  | |  |  |  | |  |
|  | $20,001-50,000 | 0.94 | 0.83 | | 1.07 | 0.94 | 0.83 | | 1.07 | 0.94 | 0.83 | | 1.07 | 0.94 | 0.83 | | 1.07 |
|  | $50,001-100,000 | 1.02 | 0.88 | | 1.18 | 1.02 | 0.88 | | 1.18 | 1.01 | 0.87 | | 1.18 | 1.01 | 0.87 | | 1.18 |
|  | $100,001+ | 0.97 | 0.81 | | 1.16 | 0.98 | 0.81 | | 1.17 | 0.97 | 0.81 | | 1.17 | 0.97 | 0.8 | | 1.16 |
| Census region (ref = Northeast) | |  |  | |  |  |  | |  |  |  | |  |  |  | |  |
|  | Midwest | 1.02 | 0.88 | | 1.18 | 1.02 | 0.88 | | 1.18 | 1.02 | 0.88 | | 1.18 | 1.02 | 0.88 | | 1.18 |
|  | South | 0.99 | 0.86 | | 1.14 | 0.99 | 0.86 | | 1.14 | 0.99 | 0.86 | | 1.14 | 0.99 | 0.86 | | 1.14 |
|  | West | 1.04 | 0.91 | | 1.19 | 1.05 | 0.92 | | 1.19 | 1.04 | 0.92 | | 1.19 | 1.04 | 0.91 | | 1.19 |
| Lifetime substance use (ref = No) | | 5.97 | 4.39 | | 8.12 | 6.03 | 4.43 | | 8.20 | 6.07 | 4.46 | | 8.25 | 5.98 | 4.39 | | 8.14 |
| Substance use disorder (ref = No) | | 1.26 | 0.97 | | 1.63 | 1.25 | 0.96 | | 1.62 | 1.29 | 0.99 | | 1.67 | 1.26 | 0.97 | | 1.64 |

aRR, adjusted risk ratio; NM ASSIST, National Institute of Drug Abuse Modified Alcohol, Smoking and Substance Involvement Screening Test; E-HITS, Extended-Hurt, Insulted, Threaten, Scream; CI, confidence interval.

**Supplemental Table 2.** Full model output of the association between any past-year intimate partner violence (2021) and NM ASSIST substance involvement scores (2022) for specific drug class among sexual and gender minority participants in The PRIDE Study

|  |  | **Cannabis** | | | **Stimulants** | | | **Hallucinogens** | | |
| --- | --- | --- | --- | --- | --- | --- | --- | --- | --- | --- |
|  |  | Adjusted *B* | 95% CI | | Adjusted *B* | 95% CI | | Adjusted *B* | 95% CI | |
| E-HITS (ref = No) | | 1.03 | 0.57 | 1.50 | 0.48 | 0.11 | 0.84 | 0.24 | 0.07 | 0.41 |
| Age, years | | -0.04 | -0.06 | -0.03 | -0.02 | -0.03 | -0.01 | -0.01 | -0.01 | 0.00 |
| Gender groups (ref = Cisgender man) | |  |  |  |  |  |  |  |  |  |
|  | Cisgender woman | 0.13 | -0.41 | 0.67 | -0.77 | -1.19 | -0.36 | -0.16 | -0.34 | 0.03 |
|  | Gender diverse AFAB | 1.07 | 0.46 | 1.68 | -0.43 | -0.89 | 0.04 | -0.15 | -0.36 | 0.05 |
|  | Gender diverse AMAB | 0.88 | -0.12 | 1.89 | -0.82 | -1.59 | -0.05 | -0.29 | -0.63 | 0.05 |
|  | Transgender man | 0.98 | 0.30 | 1.66 | -0.30 | -0.83 | 0.22 | -0.14 | -0.37 | 0.09 |
|  | Transgender woman | 0.54 | -0.30 | 1.37 | -0.65 | -1.28 | -0.01 | -0.06 | -0.34 | 0.22 |
| Sexual orientation groups (ref = Gay or Lesbian) | |  |  |  |  |  |  |  |  |  |
|  | Asexual | -1.68 | -2.81 | -0.56 | 0.13 | -0.74 | 0.99 | -0.05 | -0.43 | 0.33 |
|  | Bisexual | -0.09 | -0.81 | 0.64 | 0.01 | -0.54 | 0.57 | 0.02 | -0.23 | 0.26 |
|  | Pansexual | -0.04 | -1.21 | 1.12 | -0.16 | -1.05 | 0.74 | 0.36 | -0.04 | 0.75 |
|  | Queer | 0.51 | -0.25 | 1.27 | 0.59 | 0.01 | 1.17 | 0.45 | 0.19 | 0.70 |
|  | Heterosexual | -0.52 | -2.39 | 1.34 | 0.47 | -0.96 | 1.90 | 0.04 | -0.60 | 0.67 |
|  | Multiple options selected | -0.03 | -0.52 | 0.46 | 0.28 | -0.09 | 0.66 | 0.31 | 0.15 | 0.48 |
|  | Questioning, same-gender loving, or another sexual orientation | -2.67 | -4.90 | -0.45 | -1.11 | -2.82 | 0.60 | -0.04 | -0.79 | 0.72 |
| Education level (ref = HS or less) | |  |  |  |  |  |  |  |  |  |
|  | Some college | 0.55 | -0.42 | 1.52 | -0.01 | -0.75 | 0.74 | -0.10 | -0.42 | 0.23 |
|  | 4-year college grad | 0.14 | -0.81 | 1.09 | -0.31 | -1.03 | 0.42 | -0.20 | -0.52 | 0.12 |
|  | Advanced degree | -0.54 | -1.51 | 0.43 | -0.48 | -1.23 | 0.26 | -0.33 | -0.66 | 0.00 |
| Current Employment (ref = No) | | 0.07 | -0.38 | 0.53 | 0.01 | -0.33 | 0.36 | -0.02 | -0.18 | 0.13 |
| Individual Income (ref = $0-20,000) | |  |  |  |  |  |  |  |  |  |
|  | $20,001-50,000 | -0.01 | -0.50 | 0.48 | -0.02 | -0.40 | 0.35 | 0.13 | -0.03 | 0.30 |
|  | $50,001-100,000 | -0.07 | -0.62 | 0.49 | -0.05 | -0.48 | 0.37 | 0.06 | -0.13 | 0.25 |
|  | $100,001+ | -1.08 | -1.74 | -0.41 | -0.09 | -0.60 | 0.42 | 0.23 | 0.01 | 0.46 |
| Census region (ref = Northeast) | |  |  |  |  |  |  |  |  |  |
|  | Midwest | -0.04 | -0.58 | 0.50 | 0.12 | -0.30 | 0.53 | -0.13 | -0.32 | 0.05 |
|  | South | -0.18 | -0.69 | 0.33 | 0.11 | -0.29 | 0.50 | 0.01 | -0.17 | 0.18 |
|  | West | 0.29 | -0.19 | 0.77 | 0.15 | -0.23 | 0.52 | 0.17 | 0.01 | 0.34 |
| Lifetime substance use (ref = No) | | 3.58 | 3.05 | 4.11 | 1.37 | 0.96 | 1.77 | 0.43 | 0.25 | 0.61 |
| Substance use disorder (ref = No) | | 3.33 | 2.18 | 4.47 | 7.42 | 6.55 | 8.30 | 1.26 | 0.87 | 1.65 |
|  |  | **Opiates** | | | **Sedatives** | | | **Inhalants** | | |
|  |  | Adjusted *B* | 95% CI | | Adjusted *B* | 95% CI | | Adjusted *B* | 95% CI | |
| E-HITS (ref = No) | | 0.27 | 0.06 | 0.49 | 0.23 | -0.02 | 0.49 | 0.17 | -0.01 | 0.35 |
| Age, years | | 0.01 | 0.00 | 0.02 | 0.01 | 0.00 | 0.02 | -0.01 | -0.01 | 0.00 |
| Gender groups (ref = Cisgender man) | |  |  |  |  |  |  |  |  |  |
|  | Cisgender woman | 0.20 | -0.02 | 0.41 | 0.07 | -0.22 | 0.37 | -1.03 | -1.22 | -0.83 |
|  | Gender diverse AFAB | 0.27 | 0.03 | 0.52 | 0.20 | -0.13 | 0.53 | -0.88 | -1.09 | -0.66 |
|  | Gender diverse AMAB | -0.13 | -0.53 | 0.27 | 0.24 | -0.30 | 0.79 | -0.75 | -1.11 | -0.39 |
|  | Transgender man | 0.29 | 0.02 | 0.56 | 0.27 | -0.10 | 0.64 | -0.59 | -0.83 | -0.35 |
|  | Transgender woman | 0.39 | 0.06 | 0.72 | -0.08 | -0.53 | 0.37 | -0.77 | -1.07 | -0.47 |
| Sexual orientation groups (ref = Gay or Lesbian) | |  |  |  |  |  |  |  |  |  |
|  | Asexual | -0.28 | -0.73 | 0.16 | -0.48 | -1.09 | 0.13 | -0.23 | -0.63 | 0.17 |
|  | Bisexual | -0.15 | -0.44 | 0.13 | -0.33 | -0.72 | 0.07 | -0.14 | -0.40 | 0.12 |
|  | Pansexual | 0.08 | -0.38 | 0.55 | 0.52 | -0.11 | 1.15 | -0.16 | -0.58 | 0.26 |
|  | Queer | 0.29 | -0.01 | 0.59 | -0.22 | -0.63 | 0.19 | -0.20 | -0.47 | 0.07 |
|  | Heterosexual | -0.39 | -1.13 | 0.35 | 0.20 | -0.81 | 1.21 | -0.61 | -1.28 | 0.06 |
|  | Multiple options selected | 0.04 | -0.15 | 0.23 | -0.09 | -0.35 | 0.18 | -0.17 | -0.35 | 0.00 |
|  | Questioning, same-gender loving, or another sexual orientation | -0.63 | -1.51 | 0.25 | -0.73 | -1.94 | 0.47 | -0.47 | -1.27 | 0.33 |
| Education level (ref = HS or less) | |  |  |  |  |  |  |  |  |  |
|  | Some college | 0.19 | -0.19 | 0.57 | -0.14 | -0.66 | 0.39 | 0.00 | -0.35 | 0.34 |
|  | 4-year college grad | 0.07 | -0.31 | 0.44 | -0.30 | -0.81 | 0.22 | -0.07 | -0.41 | 0.27 |
|  | Advanced degree | 0.03 | -0.35 | 0.41 | -0.48 | -1.01 | 0.04 | -0.09 | -0.44 | 0.26 |
| Current Employment (ref = No) | | -0.09 | -0.27 | 0.09 | -0.06 | -0.31 | 0.18 | -0.09 | -0.25 | 0.07 |
| Individual Income (ref = $0-20,000) | |  |  |  |  |  |  |  |  |  |
|  | $20,001-50,000 | -0.02 | -0.22 | 0.17 | -0.19 | -0.45 | 0.08 | 0.13 | -0.05 | 0.30 |
|  | $50,001-100,000 | -0.09 | -0.31 | 0.13 | 0.00 | -0.30 | 0.30 | 0.15 | -0.05 | 0.34 |
|  | $100,001+ | -0.10 | -0.36 | 0.16 | -0.12 | -0.48 | 0.24 | 0.22 | -0.02 | 0.46 |
| Census region (ref = Northeast) | |  |  |  |  |  |  |  |  |  |
|  | Midwest | 0.03 | -0.18 | 0.24 | 0.10 | -0.19 | 0.39 | -0.01 | -0.21 | 0.19 |
|  | South | 0.04 | -0.17 | 0.24 | -0.11 | -0.38 | 0.17 | 0.11 | -0.08 | 0.29 |
|  | West | 0.11 | -0.08 | 0.30 | -0.08 | -0.34 | 0.18 | 0.02 | -0.16 | 0.19 |
| Lifetime substance use (ref = No) | | 0.37 | 0.16 | 0.58 | 1.00 | 0.72 | 1.29 | 0.40 | 0.21 | 0.59 |
| Substance use disorder (ref = No) | | 3.56 | 3.11 | 4.01 | 1.81 | 1.19 | 2.43 | 1.11 | 0.70 | 1.52 |

NM ASSIST, National Institute of Drug Abuse Modified Alcohol, Smoking and Substance Involvement Screening Test; E-HITS, Extended-Hurt, Insulted, Threaten, Scream; CI, confidence interval.

**Supplemental Table 3.** Full model output of the association between intimate partner violence frequency (E-HITS scores, 2021) and NM ASSIST substance involvement scores (2022) for specific drug class among sexual and gender minority participants in The PRIDE Study

|  |  | **Cannabis** | | | **Stimulants** | | | **Hallucinogens** | | |
| --- | --- | --- | --- | --- | --- | --- | --- | --- | --- | --- |
|  |  | Adjusted *B* | 95% CI | | Adjusted *B* | 95% CI | | Adjusted *B* | 95% CI | |
| E-HITS scores | | 0.22 | 0.08 | 0.36 | 0.20 | 0.06 | 0.35 | 0.07 | 0.02 | 0.12 |
| Age, years | | -0.04 | -0.06 | -0.03 | -0.02 | -0.03 | -0.01 | -0.01 | -0.01 | 0.00 |
| Gender groups (ref = Cisgender man) | |  |  |  |  |  |  |  |  |  |
|  | Cisgender woman | 0.16 | -0.39 | 0.70 | -0.75 | -1.17 | -0.34 | -0.15 | -0.33 | 0.03 |
|  | Gender diverse AFAB | 1.07 | 0.46 | 1.68 | -0.43 | -0.90 | 0.04 | -0.15 | -0.36 | 0.05 |
|  | Gender diverse AMAB | 0.91 | -0.10 | 1.92 | -0.81 | -1.58 | -0.04 | -0.28 | -0.62 | 0.06 |
|  | Transgender man | 0.97 | 0.29 | 1.65 | -0.32 | -0.84 | 0.21 | -0.15 | -0.38 | 0.09 |
|  | Transgender woman | 0.52 | -0.31 | 1.35 | -0.67 | -1.31 | -0.03 | -0.06 | -0.34 | 0.22 |
| Sexual orientation groups (ref = Gay or Lesbian) | |  |  |  |  |  |  |  |  |  |
|  | Asexual | -1.74 | -2.87 | -0.62 | 0.12 | -0.74 | 0.99 | -0.06 | -0.44 | 0.32 |
|  | Bisexual | -0.10 | -0.83 | 0.63 | 0.01 | -0.55 | 0.56 | 0.02 | -0.23 | 0.26 |
|  | Pansexual | -0.02 | -1.19 | 1.14 | -0.17 | -1.06 | 0.72 | 0.36 | -0.03 | 0.76 |
|  | Queer | 0.50 | -0.26 | 1.26 | 0.59 | 0.01 | 1.17 | 0.45 | 0.19 | 0.70 |
|  | Heterosexual | -0.48 | -2.35 | 1.39 | 0.46 | -0.97 | 1.89 | 0.04 | -0.59 | 0.67 |
|  | Multiple options selected | -0.02 | -0.51 | 0.47 | 0.30 | -0.08 | 0.67 | 0.32 | 0.15 | 0.48 |
|  | Questioning, same-gender loving, or another sexual orientation | -2.73 | -4.96 | -0.50 | -1.16 | -2.86 | 0.55 | -0.05 | -0.81 | 0.70 |
| Education level (ref = HS or less) | |  |  |  |  |  |  |  |  |  |
|  | Some college | 0.52 | -0.45 | 1.49 | -0.02 | -0.76 | 0.72 | -0.10 | -0.43 | 0.22 |
|  | 4-year college grad | 0.11 | -0.84 | 1.06 | -0.29 | -1.02 | 0.43 | -0.20 | -0.52 | 0.12 |
|  | Advanced degree | -0.57 | -1.54 | 0.40 | -0.48 | -1.22 | 0.27 | -0.33 | -0.66 | -0.01 |
| Current Employment (ref = No) | | 0.09 | -0.36 | 0.54 | 0.04 | -0.31 | 0.38 | -0.02 | -0.17 | 0.14 |
| Individual Income (ref = $0-20,000) | |  |  |  |  |  |  |  |  |  |
|  | $20,001-50,000 | 0.00 | -0.49 | 0.49 | -0.01 | -0.39 | 0.36 | 0.13 | -0.03 | 0.30 |
|  | $50,001-100,000 | -0.05 | -0.61 | 0.51 | -0.04 | -0.46 | 0.39 | 0.07 | -0.12 | 0.25 |
|  | $100,001+ | -1.04 | -1.70 | -0.37 | -0.07 | -0.58 | 0.44 | 0.24 | 0.02 | 0.47 |
| Census region (ref = Northeast) | |  |  |  |  |  |  |  |  |  |
|  | Midwest | -0.05 | -0.59 | 0.49 | 0.09 | -0.32 | 0.50 | -0.14 | -0.32 | 0.04 |
|  | South | -0.19 | -0.70 | 0.32 | 0.10 | -0.29 | 0.49 | 0.01 | -0.17 | 0.18 |
|  | West | 0.29 | -0.20 | 0.77 | 0.14 | -0.23 | 0.51 | 0.17 | 0.01 | 0.34 |
| Lifetime substance use (ref = No) | | 3.62 | 3.08 | 4.15 | 1.36 | 0.95 | 1.77 | 0.43 | 0.25 | 0.61 |
| Substance use disorder (ref = No) | | 3.30 | 2.15 | 4.45 | 7.30 | 6.42 | 8.18 | 1.24 | 0.85 | 1.63 |
|  |  | **Opiates** | | | **Sedatives** | | | **Inhalants** | | |
|  |  | Adjusted *B* | 95% CI | | Adjusted *B* | 95% CI | | Adjusted *B* | 95% CI | |
| E-HITS scores | | 0.12 | 0.03 | 0.20 | 0.04 | -0.04 | 0.12 | 0.03 | -0.01 | 0.10 |
| Age, years | | 0.01 | 0.01 | 0.02 | 0.01 | 0.00 | 0.02 | -0.01 | -0.01 | 0.00 |
| Gender groups (ref = Cisgender man) | |  |  |  |  |  |  |  |  |  |
|  | Cisgender woman | 0.20 | -0.01 | 0.42 | 0.08 | -0.21 | 0.38 | -0.15 | -0.33 | 0.03 |
|  | Gender diverse AFAB | 0.27 | 0.03 | 0.51 | 0.20 | -0.13 | 0.53 | -0.15 | -0.36 | 0.05 |
|  | Gender diverse AMAB | -0.13 | -0.52 | 0.27 | 0.26 | -0.29 | 0.80 | -0.28 | -0.62 | 0.06 |
|  | Transgender man | 0.29 | 0.02 | 0.56 | 0.27 | -0.10 | 0.64 | -0.15 | -0.38 | 0.09 |
|  | Transgender woman | 0.38 | 0.05 | 0.71 | -0.09 | -0.54 | 0.36 | -0.06 | -0.34 | 0.22 |
| Sexual orientation groups (ref = Gay or Lesbian) | |  |  |  |  |  |  |  |  |  |
|  | Asexual | -0.28 | -0.72 | 0.16 | -0.51 | -1.12 | 0.10 | -0.06 | -0.44 | 0.32 |
|  | Bisexual | -0.16 | -0.44 | 0.13 | -0.33 | -0.73 | 0.06 | 0.02 | -0.23 | 0.26 |
|  | Pansexual | 0.08 | -0.38 | 0.54 | 0.53 | -0.10 | 1.16 | 0.36 | -0.03 | 0.76 |
|  | Queer | 0.29 | -0.01 | 0.59 | -0.22 | -0.63 | 0.19 | 0.45 | 0.19 | 0.70 |
|  | Heterosexual | -0.39 | -1.13 | 0.34 | 0.21 | -0.80 | 1.22 | 0.04 | -0.59 | 0.67 |
|  | Multiple options selected | 0.05 | -0.15 | 0.24 | -0.08 | -0.35 | 0.18 | 0.32 | 0.15 | 0.48 |
|  | Questioning, same-gender loving, or another sexual orientation | -0.65 | -1.53 | 0.23 | -0.76 | -1.97 | 0.45 | -0.05 | -0.81 | 0.70 |
| Education level (ref = HS or less) | |  |  |  |  |  |  |  |  |  |
|  | Some college | 0.18 | -0.20 | 0.57 | -0.15 | -0.68 | 0.38 | -0.10 | -0.43 | 0.22 |
|  | 4-year college grad | 0.08 | -0.30 | 0.45 | -0.30 | -0.82 | 0.21 | -0.20 | -0.52 | 0.12 |
|  | Advanced degree | 0.04 | -0.35 | 0.42 | -0.49 | -1.02 | 0.03 | -0.33 | -0.66 | -0.01 |
| Current Employment (ref = No) | | -0.08 | -0.26 | 0.10 | -0.06 | -0.30 | 0.19 | -0.02 | -0.17 | 0.14 |
| Individual Income (ref = $0-20,000) | |  |  |  |  |  |  |  |  |  |
|  | $20,001-50,000 | -0.02 | -0.21 | 0.17 | -0.18 | -0.45 | 0.08 | 0.13 | -0.03 | 0.30 |
|  | $50,001-100,000 | -0.08 | -0.30 | 0.14 | 0.00 | -0.30 | 0.30 | 0.07 | -0.12 | 0.25 |
|  | $100,001+ | -0.09 | -0.35 | 0.17 | -0.10 | -0.46 | 0.26 | 0.24 | 0.02 | 0.47 |
| Census region (ref = Northeast) | |  |  |  |  |  |  |  |  |  |
|  | Midwest | 0.02 | -0.19 | 0.23 | 0.10 | -0.20 | 0.39 | -0.14 | -0.32 | 0.04 |
|  | South | 0.03 | -0.17 | 0.24 | -0.11 | -0.38 | 0.17 | 0.01 | -0.17 | 0.18 |
|  | West | 0.11 | -0.08 | 0.30 | -0.08 | -0.34 | 0.18 | 0.17 | 0.01 | 0.34 |
| Lifetime substance use (ref = No) | | 0.37 | 0.16 | 0.58 | 1.02 | 0.73 | 1.30 | 0.43 | 0.25 | 0.61 |
| Substance use disorder (ref = No) | | 3.50 | 3.05 | 3.95 | 1.79 | 1.17 | 2.41 | 1.24 | 0.85 | 1.63 |

Overall E-HITS scores were mean centered. NM ASSIST, National Institute of Drug Abuse Modified Alcohol, Smoking and Substance Involvement Screening Test; E-HITS, Extended-Hurt, Insulted, Threaten, Scream; CI, confidence interval.

**Supplemental Table 4.** Participants characteristics by current relationship status

|  | In a current relationship | |
| --- | --- | --- |
|  | No  (n = 1250) | Yes  (n = 2470) |
| Age, years (median, IQR) | 32.1 (25.6-47.7) | 35.3 (28.7-48.6) |
| Gender groups (n, %) |  |  |
| Cisgender man | 315 (25.2) | 600 (24.3) |
| Cisgender woman | 267 (21.4) | 710 (28.7) |
| Gender diverse, assigned female at birth | 330 (26.4) | 604 (24.5) |
| Gender diverse, assigned male at birth | 52 (4.2) | 86 (3.5) |
| Transgender man | 213 (17.0) | 321 (13.0) |
| Transgender woman | 73 (5.8) | 149 (6.0) |
| Sexual orientation groups (n, %) |  |  |
| Asexual | 80 (6.4) | 27 (1.1) |
| Bisexual | 98 (7.8) | 203 (8.2) |
| Gay or Lesbian | 356 (28.5) | 749 (30.3) |
| Pansexual | 27 (2.2) | 66 (2.7) |
| Queer | 85 (6.8) | 219 (8.9) |
| Heterosexual | 3 (0.2) | 31 (1.3) |
| Multiple options selected | 593 (47.4) | 1160 (47.0) |
| Questioning, same-gender loving, or another sexual orientation | 8 (0.6) | 15 (0.6) |
| Ethnoracial identity^a,b^ (n, %) |  |  |
| American Indian or Alaska Native | 32 (2.6) | 72 (2.9) |
| Asian | 67 (5.4) | 101 (4.1) |
| Black, African American or African | 46 (3.7) | 90 (3.6) |
| Hispanic, Latino or Spanish | 74 (5.9) | 148 (6.0) |
| Middle Eastern or North African | 20 (1.6) | 34 (1.4) |
| Native Hawaiian or other Pacific Islander | 3 (0.2) | 5 (0.2) |
| White | 1145 (91.6) | 2282 (92.4) |
| Another | 20 (1.6) | 36 (1.5) |
| Education level (n, %) |  |  |
| High school or less | 75 (6.0) | 65 (2.6) |
| Some college | 289 (23.1) | 398 (16.1) |
| 4-year college grad | 467 (37.4) | 836 (33.8) |
| Advanced degree | 418 (33.4) | 1171 (47.4) |
| Missing | 1 (0.1) | 0 (0.0) |
| Employment (n, %) |  |  |
| No | 411 (32.9) | 577 (23.4) |
| Yes | 838 (67.0) | 1892 (76.6) |
| Missing | 1 (0.1) | 1 (0.0) |
| Individual Income (n, %) |  |  |
| $0-20,000 | 484 (38.7) | 585 (23.7) |
| $20,001-50,000 | 383 (30.6) | 712 (28.8) |
| $50,001-100,000 | 249 (19.9) | 728 (29.5) |
| $100,001+ | 124 (9.9) | 429 (17.4) |
| Missing | 10 (0.8) | 16 (0.6) |
| Region (n, %) |  |  |
| Northeast | 252 (20.2) | 509 (20.6) |
| Midwest | 244 (19.5) | 513 (20.8) |
| South | 341 (27.3) | 604 (24.5) |
| West | 398 (31.8) | 836 (33.8) |
| Missing | 15 (1.2) | 8 (0.3) |
| E-HITS scores (mean, SD) |  |  |
| Any | 5.45 (1.64) | 5.74 (1.52) |
| Physical | 2.07 (0.44) | 2.06 (0.37) |
| Sexual | 1.04 (0.32) | 1.03 (0.26) |
| Emotional | 2.33 (1.15) | 2.65 (1.25) |
| Lifetime substance use (n, %) |  |  |
| No | 242 (19.4) | 228 (9.2) |
| Yes | 990 (79.2) | 2204 (89.2) |
| Missing | 18 (1.4) | 38 (1.5) |
| Past-year substance use (n, %) |  |  |
| No | 527 (42.2) | 849 (34.4) |
| Yes | 705 (56.4) | 1583 (64.1) |
| Missing | 18 (1.4) | 38 (1.5) |
| Substance use disorder diagnosis (n, %) | 38 (3.0) | 48 (1.9) |
| Substance-specific ASSIST scores (mean, SD) |  |  |
| Cannabis | 3.15 (5.70) | 3.59 (5.69) |
| Stimulants | 1.79 (5.29) | 1.62 (4.00) |
| Hallucinogens | 0.44 (1.92) | 0.52 (1.98) |
| Narcotics | 0.57 (2.41) | 0.63 (2.38) |
| Sedatives | 1.25 (3.31) | 1.09 (2.90) |
| Inhalants | 0.46 (1.82) | 0.50 (2.13) |

^a^ Participants could select multiple responses; thus, the sum of percentages is greater than 100%.

^b^ About 10.5% selected multiple ethnoracial identities.

IQR, interquartile range; AFAB, assigned female at birth; AMAB, assigned male at birth; E-HITS, Extended-Hurt, Insulted, Threaten, Scream.

**Supplemental Table 5.** Association between intimate partner violence and its subtypes with any past 30-day substance use among sexual and gender minority participants who are currently in a relationship in The PRIDE Study, 2021-2022

| Exposure Type |  | Total | Any past 30-day Substance Use | Unadjusted RR  (95% CI) | Adjusted RR  (95% CI) |
| --- | --- | --- | --- | --- | --- |
|  |  | (n = 2470) | (n = 1225) |  |  |
|  | Exposure | n (%) | n (Row %) |  |  |
| Overall | E-HITS |  |  |  |  |
|  | No | 1699 (68.8) | 808 (48.4) | Ref | Ref |
|  | Yes | 771 (31.2) | 417 (54.1) | **1.14 (1.05, 1.23)** | 1.08 (0.99, 1.18) |
| Type of E-HITS | Physical |  |  |  |  |
|  | No | 2384 (96.5) | 1167 (49.0) | Ref | Ref |
|  | Yes | 86 (3.5) | 58 (67.4) | **1.38 (1.18, 1.60)** | **1.24 (1.06, 1.46)** |
|  | Sexual |  |  |  |  |
|  | No | 2418 (97.9) | 1198 (49.5) | Ref | Ref |
|  | Yes | 52 (2.1) | 27 (51.9) | 1.05 (0.80, 1.37) | 0.85 (0.64, 1.13) |
|  | Emotional |  |  |  |  |
|  | No | 1720 (69.9) | 819 (47.6) | Ref | Ref |
|  | Yes | 750 (30.4) | 406 (54.1) | **1.14 (1.05, 1.23)** | 1.09 (1.00, 1.19) |

Models adjusted for age (continuous), gender identity, sexual orientation, education, employment, income, Census region, lifetime substance use, substance use disorder, and current relationship satisfaction. E-HITS, Extended-Hurt, Insulted, Threaten, Scream; RR, risk ratio; CI, confidence interval. Bolded estimates indicate p<0.05.

**Supplemental Table 6.** Association between any past-year intimate partner violence and NM ASSIST substance involvement scores for specific drug class among sexual and gender minority participants who are currently in a relationship in The PRIDE Study, 2021-2022

| ASSIST Scores | E-HITS | | Unadjusted *B*  (95% CI) | Adjusted *B*  (95% CI) |
| --- | --- | --- | --- | --- |
|  | No  (n = 1699) | Yes  (n = 771) |  |  |
|  | Mean (SD) | Mean (SD) |  |  |
| Cannabis | 3.28 (5.38) | 4.25 (6.27) | **0.97 (0.45, 1.48)** | **1.00 (0.49, 1.50)** |
| Stimulants | 1.37 (3.49) | 2.16 (4.89) | **0.79 (0.41, 1.17)** | **0.47 (0.11, 0.83)** |
| Hallucinogens | 0.44 (1.74) | 0.71 (2.42) | **0.27 (0.08, 0.46)** | **0.19 (0.02, 0.36)** |
| Opiates | 0.51 (2.06) | 0.88 (2.97) | **0.37 (0.14, 0.60)** | 0.22 (0.00, 0.45) |
| Sedatives | 0.92 (2.73) | 1.44 (3.23) | **0.52 (0.25, 0.78)** | 0.25 (-0.02, 0.53) |
| Inhalants | 0.45 (1.94) | 0.63 (2.51) | 0.18 (-0.02, 0.38) | 0.05 (-0.14, 0.25) |

Models adjusted for age (continuous), gender identity, sexual orientation, education, employment, income, Census region, lifetime substance use, substance use disorder, and current relationship satisfaction. NM ASSIST, National Institute of Drug Abuse Modified Alcohol, Smoking and Substance Involvement Screening Test; E-HITS, Extended-Hurt, Insulted, Threaten, Scream; CI, confidence interval. Bolded estimates indicate p<0.05.

**Supplemental Table 7.** Association between intimate partner violence severity (E-HITS scores) and NM ASSIST substance involvement scores for specific drug class among sexual and gender minority participants who are currently in a relationship in The PRIDE Study, 2021-2022

| ASSIST Scores | Unadjusted *B*  (95% CI) | Adjusted *B*  (95% CI) |
| --- | --- | --- |
| Cannabis | **0.28 (0.11, 0.45)** | **0.26 (0.08, 0.43)** |
| Stimulants | **0.39 (0.20, 0.58)** | **0.29 (0.11, 0.48)** |
| Hallucinogens | **0.11 (0.04, 0.18)** | **0.09 (0.01, 0.16)** |
| Opiates | **0.17 (0.06, 0.28)** | **0.14 (0.03, 0.25)** |
| Sedatives | **0.15 (0.05, 0.24)** | 0.07 (-0.03, 0.16) |
| Inhalants | **0.08 (0.01, 0.16)** | 0.03 (-0.02, 0.11) |

Models adjusted for age (continuous), gender identity, sexual orientation, education, employment, income, Census region, lifetime substance use, substance use disorder, and current relationship satisfaction. Overall E-HITS scores were mean centered. NM ASSIST, National Institute of Drug Abuse Modified Alcohol, Smoking and Substance Involvement Screening Test; E-HITS, Extended-Hurt, Insulted, Threaten, Scream; CI, confidence interval.

**Supplemental Table 8.** Association between intimate partner violence and past 30-day substance use by gender groups among sexual and gender minority participants in The PRIDE Study, 2021-2022

| Gender groups | Any IPV exposure | Total | Past 30-day Substance Use | Adjusted RR  (95% CI) |
| --- | --- | --- | --- | --- |
|  |  | (n = 3745) | (n = 1803) |  |
|  |  | n (%) | n (Row %) |  |
| Cisgender man | No | 683 (74.2) | 277 (40.6) | Ref |
|  | Yes | 238 (25.8) | 122 (51.3) | **1.18 (1.02, 1.36)** |
| Cisgender woman | No | 730 (74.3) | 293 (40.1) | Ref |
|  | Yes | 252 (25.7) | 126 (50.0) | **1.17 (1.01, 1.36)** |
| Gender diverse AFAB | No | 737 (78.2) | 378 (51.3) | Ref |
|  | Yes | 206 (21.8) | 124 (60.2) | 1.07 (0.94, 1.21) |
| Gender diverse AMAB | No | 99 (71.7) | 52 (52.5) | Ref |
|  | Yes | 39 (28.3) | 19 (48.7) | 0.85 (0.59, 1.21) |
| Transgender man | No | 409 (75.9) | 225 (55.0) | Ref |
|  | Yes | 130 (24.1) | 82 (63.1) | 1.11 (0.95, 1.29) |
| Transgender woman | No | 165 (74.3) | 70 (42.4) | Ref |
|  | Yes | 57 (25.7) | 35 (61.4) | 1.23 (0.96, 1.59) |

Models adjusted for age (continuous), sexual orientation groups, education, employment, income, Census region, lifetime substance use, substance use disorder, and current relationship satisfaction. E-HITS, Extended-Hurt, Insulted, Threaten, Scream; RR, risk ratio; CI, confidence interval; AFAB, assigned female at birth; AMAB, assigned male at birth. Bolded estimates indicate p<0.05.

**Supplemental Table 9.** Association between past-year intimate partner violence and NM ASSIST substance involvement scores for specific drug class by gender groups among sexual and gender minority participants in The PRIDE Study, 2021-2022

| Substance Involvement Scores by Gender Groups | E-HITS | | Adjusted *B*  (95% CI) |
| --- | --- | --- | --- |
|  | No  (n = 2823) | Yes  (n = 922) |  |
|  | Mean (SD) | Mean (SD) |  |
| Cisgender man |  |  |  |
| Cannabis | 2.1 (4.1) | 2.9 (4.9) | 0.50 (-0.18, 1.18) |
| Stimulants | 1.3 (4.3) | 3.1 (8.9) | **1.48 (0.44, 2.52)** |
| Hallucinogens | 0.3 (1.3) | 0.7 (2.4) | 0.29 (-0.03, 0.61) |
| Narcotics | 0.3 (1.5) | 0.9 (3.5) | **0.49 (0.05, 0.94)** |
| Sedatives | 1.0 (2.9) | 1.5 (3.7) | 0.44 (-0.07, 0.96) |
| Inhalants | 1.1 (2.9) | 1.3 (3.1) | 0.14 (-0,.31, 0.59) |
| Cisgender woman |  |  |  |
| Cannabis | 2.6 (4.7) | 4.0 (5.9) | **1.17 (0.41, 1.92)** |
| Stimulants | 1.0 (2.8) | 1.7 (3.9) | 0.48 (-0.05, 1.01) |
| Hallucinogens | 0.3 (1.5) | 0.7 (3.1) | 0.29 (-0.10, 0.68) |
| Narcotics | 0.4 (1.5) | 1.0 (2.8) | **0.44 (0.10, 0.78)** |
| Sedatives | 0.9 (2.8) | 1.3 (3.1) | 0.24 (-0.19, 0.68) |
| Inhalants | 0.1 (0.6) | 0.2 (0.8) | 0.05 (-0.07, 0.16) |
| Gender diverse AFAB |  |  |  |
| Cannabis | 4.0 (6.4) | 5.1 (7.1) | 0.73 (-0.32, 1.78) |
| Stimulants | 1.6 (3.6) | 2.1 (3.8) | 0.36 (-0.22, 0.95) |
| Hallucinogens | 0.5 (1.7) | 0.7 (1.9) | 0.16 (-0.13, 0.45) |
| Narcotics | 0.6 (1.9) | 0.6 (2.2) | -0.02 (-0.36, 0.31) |
| Sedatives | 0.9 (2.4) | 1.5 (3.1) | **0.46 (0.01, 0.92)** |
| Inhalants | 0.2 (1.1) | 0.3 (1.2) | 0.07 (-0.12, 0.26) |
| Gender diverse AMAB |  |  |  |
| Cannabis | 3.6 (5.9) | 4.5 (5.4) | 0.44 (-1.52, 2.39) |
| Stimulants | 1.5 (3.8) | 1.1 (2.9) | -0.70 (-1.75, 0.35) |
| Hallucinogens | 0.2 (0.9) | 0.7 (1.9) | 0.45 (-0.15, 1.05) |
| Narcotics | 0.3 (1.2) | 0.5 (1.3) | 0.04 (-0.47, 0.56) |
| Sedatives | 1.2 (3.2) | 1.4 (3.1) | 0.12 (-1.01, 1.25) |
| Inhalants | 0.1 (0.6) | 0.9 (2.4) | **0.79 (0.05, 1.52)** |
| Transgender man |  |  |  |
| Cannabis | 4.1 (6.4) | 5.4 (7.1) | 1.19 (-0.14, 2.52) |
| Stimulants | 1.8 (4.1) | 2.7 (5.3) | 0.66 (-0.30, 1.63) |
| Hallucinogens | 0.6 (2.1) | 0.6 (1.6) | -0.01 (-0.34, 0.32) |
| Narcotics | 0.6 (2.3) | 0.9 (2.6) | 0.24 (-0.26, 0.74) |
| Sedatives | 1.1 (3.1) | 1.7 (3.1) | 0.39 (-0.23, 1.00) |
| Inhalants | 0.4 (1.7) | 1.0 (4.1) | 0.62 (-0.09, 1.32) |
| Transgender woman |  |  |  |
| Cannabis | 2.6 (4.4) | 4.3 (5.7) | 1.13 (-0.39, 2.64) |
| Stimulants | 1.1 (2.9) | 1.5 (4.2) | 0.17 (-0.97, 1.30) |
| Hallucinogens | 0.4 (1.6) | 1.0 (2.6) | 0.52 (-0.20, 1.23) |
| Narcotics | 0.8 (4.0) | 0.9 (2.3) | 0.08 (-0.74, 0.90) |
| Sedatives | 0.8 (3.1) | 1.4 (3.4) | 0.48 (-0.50, 1.46) |
| Inhalants | 0.3 (1.1) | 0.2 (1.1) | -0.12 (-0.47, 0.23) |

Models adjusted for age (continuous), sexual orientation groups, education, employment, income, Census region, lifetime substance use, substance use disorder, and current relationship satisfaction. NM ASSIST, National Institute of Drug Abuse Modified Alcohol, Smoking and Substance Involvement Screening Test; E-HITS, Extended-Hurt, Insulted, Threaten, Scream; CI, confidence interval; AFAB, assigned female at birth; AMAB, assigned male at birth. Bolded estimates indicate p<0.05.

**Supplemental Table 10.** Association between past-year intimate partner violence (2021) and NM ASSIST substance involvement scores (2022), using the maximum and mean substance involvement score within each drug class, among sexual and gender minority participants in The PRIDE Study

|  |  | E-HITS | | | | Unadjusted *B*  (95% CI) | Adjusted *B*  (95% CI) |
| --- | --- | --- | --- | --- | --- | --- | --- |
|  | Total  (n = 3745) | No  (n = 2823) | | | Yes  (n = 922) |  |  |
|  | Mean (SD) | Mean (SD) | | | Mean (SD) |  |  |
| **Maximum ASSIST Scores** | | | | | | | |
| Cannabis | 3.46 (5.73) | 3.18 (5.50) | | | 4.32 (6.30) | **1.15 (0.69, 1.60)** | **1.03 (0.57, 1.50)** |
| Stimulants | 1.43 (3.68) | 1.25 (3.29) | | | 1.98 (4.63) | **0.73 (0.40, 1.05)** | **0.55 (0.26, 0.85)** |
| Hallucinogens | 0.41 (1.54) | 0.35 (1.41) | | | 0.58 (2.09) | **0.24 (0.10, 0.37)** | **0.18 (0.06, 0.32)** |
| Opiates | 0.53 (1.94) | 0.44 (1.72) | | | 0.80 (2.48) | **0.36 (0.19, 0.53)** | **0.25 (0.08, 0.42)** |
| Sedatives | 1.08 (2.91) | 0.95 (2.79) | | | 1.46 (3.24) | **0.51 (0.27, 0.74)** | **0.35 (0.12, 0.59)** |
| Inhalants | 0.44 (1.79) | 0.40 (1.67) | | | 0.58 (2.09) | **0.19 (0.04, 0.34)** | 0.13 (-0.01, 0.28) |
| **Mean ASSIST Scores** | | | | | | | |
| Cannabis | 3.46 (5.73) | | 3.18 (5.50) | 4.32 (6.30) | | **1.15 (0.69, 1.60)** | **1.03 (0.57, 1.50)** |
| Stimulants | 0.53 (1.43) | | 0.46 (1.22) | 0.75 (1.92) | | **0.30 (0.16, 0.43)** | **0.22 (0.10, 0.34)** |
| Hallucinogens | 0.23 (0.98) | | 0.19 (0.80) | 0.34 (1.21) | | **0.15 (0.07, 0.23)** | **0.12 (0.04, 0.20)** |
| Opiates | 0.28 (1.10) | | 0.23 (0.99) | 0.43 (1.39) | | **0.20 (0.10, 0.30)** | **0.14 (0.04, 0.24)** |
| Sedatives | 0.54 (1.47) | | 0.48 (1.40) | 0.74 (1.65) | | **0.27 (0.15, 0.38)** | **0.19 (0.07, 0.31)** |
| Inhalants | 0.24 (0.98) | | 0.21 (0.89) | 0.32 (1.21) | | **0.12 (0.03, 0.20)** | **0.09 (0.01, 0.17)** |

Models adjusted for age (continuous), gender identity, sexual orientation, education, employment, income, Census region, lifetime substance use, and substance use disorder. NM ASSIST, National Institute of Drug Abuse Modified Alcohol, Smoking and Substance Involvement Screening Test; E-HITS, Extended-Hurt, Insulted, Threaten, Scream; CI, confidence interval. Bolded estimates indicate p<0.05.

For each substance, a total score was calculated by summing responses to relevant ASSIST items. These substance-specific scores were then grouped into broader drug classes (*i.e.,* stimulants, hallucinogens, opiates, sedatives, and inhalants) using two summary measures: (1) the maximum score among substances within each class (max ASSIST score), and (2) the mean score across substances within each class (mean ASSIST score).

**Supplemental Table 11.** Association between intimate partner violence frequency (E-HITS scores, 2021) and NM ASSIST substance involvement scores (2022), using the maximum and mean substance involvement score within each drug class, among sexual and gender minority participants in The PRIDE Study

|  | Unadjusted *B*  (95% CI) | Adjusted *B*  (95% CI) |
| --- | --- | --- |
| **Maximum ASSIST Scores** | | |
| Cannabis | **0.28 (0.15, 0.41)** | **0.22 (0.08, 0.36)** |
| Stimulants | **0.29 (0.15, 0.42)** | **0.21 (0.08, 0.35)** |
| Hallucinogens | **0.06 (0.03, 0.10)** | **0.04 (0.01, 0.08)** |
| Opiates | **0.14 (0.07, 0.20)** | **0.10 (0.03, 0.16)** |
| Sedatives | **0.12 (0.05, 0.18)** | **0.07 (0.01, 0.14)** |
| Inhalants | **0.06 (0.01, 0.10)** | **0.04 (0.00, 0.08)** |
| **Mean ASSIST Scores** | | |
| Cannabis | **0.28 (0.15, 0.41)** | **0.22 (0.08, 0.36)** |
| Stimulants | **0.11 (0.06, 0.16)** | **0.08 (0.03, 0.13)** |
| Hallucinogens | **0.04 (0.02, 0.06)** | **0.03 (0.01, 0.05)** |
| Opiates | **0.08 (0.04, 0.11)** | **0.05 (0.02, 0.09)** |
| Sedatives | **0.06 (0.03, 0.10)** | **0.04 (0.00, 0.08)** |
| Inhalants | **0.04 (0.01, 0.06)** | **0.02 (0.00, 0.05)** |

Models adjusted for age (continuous), gender identity, sexual orientation, education, employment, income, Census region, lifetime substance use, and substance use disorder. NM ASSIST, National Institute of Drug Abuse Modified Alcohol, Smoking and Substance Involvement Screening Test; E-HITS, Extended-Hurt, Insulted, Threaten, Scream; CI, confidence interval. Bolded estimates indicate p<0.05.

For each substance, a total score was calculated by summing responses to relevant ASSIST items. These substance-specific scores were then grouped into broader drug classes (*i.e.,* stimulants, hallucinogens, opiates, sedatives, and inhalants) using two summary measures: (1) the maximum score among substances within each class (max ASSIST score), and (2) the mean score across substances within each class (mean ASSIST score).

**Supplemental Table 12.** Association between intimate partner violence and its subtypes (2021) with past 30-day substance use (2022) adjusting for past year substance use in 2021 among sexual and gender minority participants in The PRIDE Study, 2021-2022

| **Exposure Type** | Adjusted RR (95% CI) |
| --- | --- |
| Overall E-HITS (ref = No) | 1.03 (0.97, 1.10) |
| Physical E-HITS (ref = No) | 1.12 (1.00, 1.25) |
| Sexual E-HITS (ref = No) | 0.89 (0.75, 1.06) |
| Emotional E-HITS (ref = No) | 1.04 (0.98, 1.11) |

Models were also adjusted for age (continuous), gender identity, sexual orientation, education level, employment status, income, Census region, and substance use disorder. E-HITS, Extended-Hurt, Insulted, Threaten, Scream; RR, risk ratio; CI, confidence interval. Bolded estimates indicate p<0.05.

**Supplemental Table 13.** Association between intimate partner violence (2021) and NM ASSIST substance involvement scores (2022) adjusting for past year substance use in 2021 among sexual and gender minority participants in The PRIDE Study, 2021-2022

| ASSIST Scores | **Past Year E-HITS (ref = No)** | **E-HITS Scores** |
| --- | --- | --- |
|  | Adjusted *B*  (95% CI) | Adjusted *B*  (95% CI) |
| Cannabis | **0.58 (0.17, 0.99)** | 0.10 (-0.02, 0.23) |
| Stimulants | **0.55 (0.19, 0.92)** | **0.21 (0.06, 0.36)** |
| Hallucinogens | **0.20 (0.04, 0.37)** | **0.05 (0.01, 0.10)** |
| Opiates | **0.24 (0.05, 0.43)** | **0.10 (0.02, 0.17)** |
| Sedatives | **0.28 (0.04, 0.52)** | 0.06 (-0.01, 0.14) |
| Inhalants | 0.13 (-0.03, 0.28) | 0.04 (-0.01, 0.09) |

Both sets of models were adjusted for age (continuous), gender identity, sexual orientation, education level, employment status, income, Census region, and substance use disorder. Overall E-HITS scores were mean centered. NM ASSIST, National Institute of Drug Abuse Modified Alcohol, Smoking and Substance Involvement Screening Test; E-HITS, Extended-Hurt, Insulted, Threaten, Scream; CI, confidence interval. Bolded estimates indicate p<0.05.
